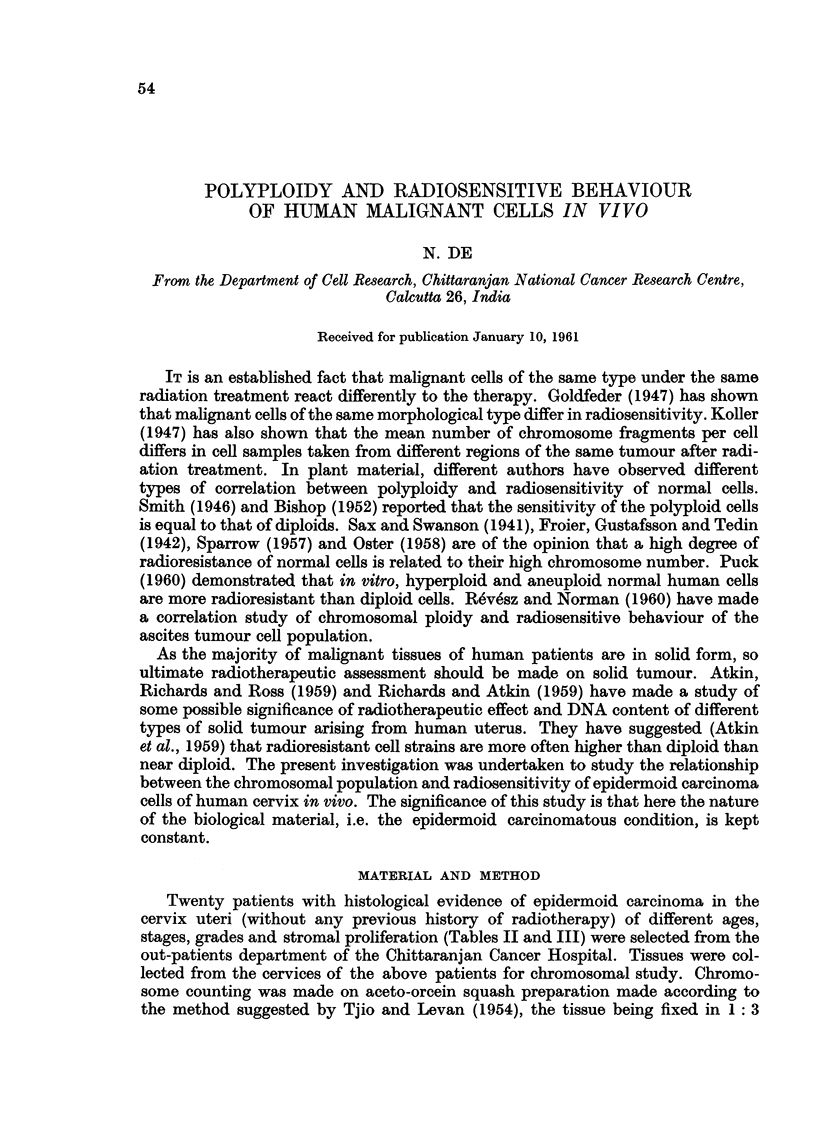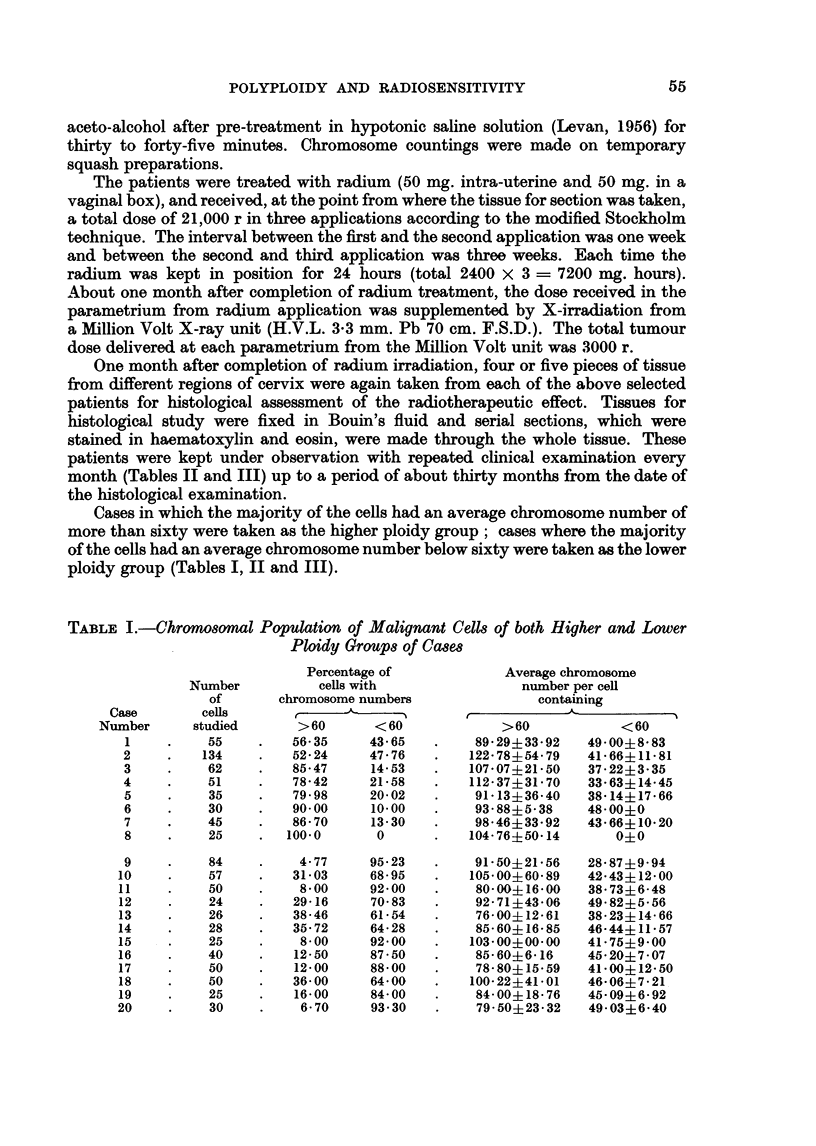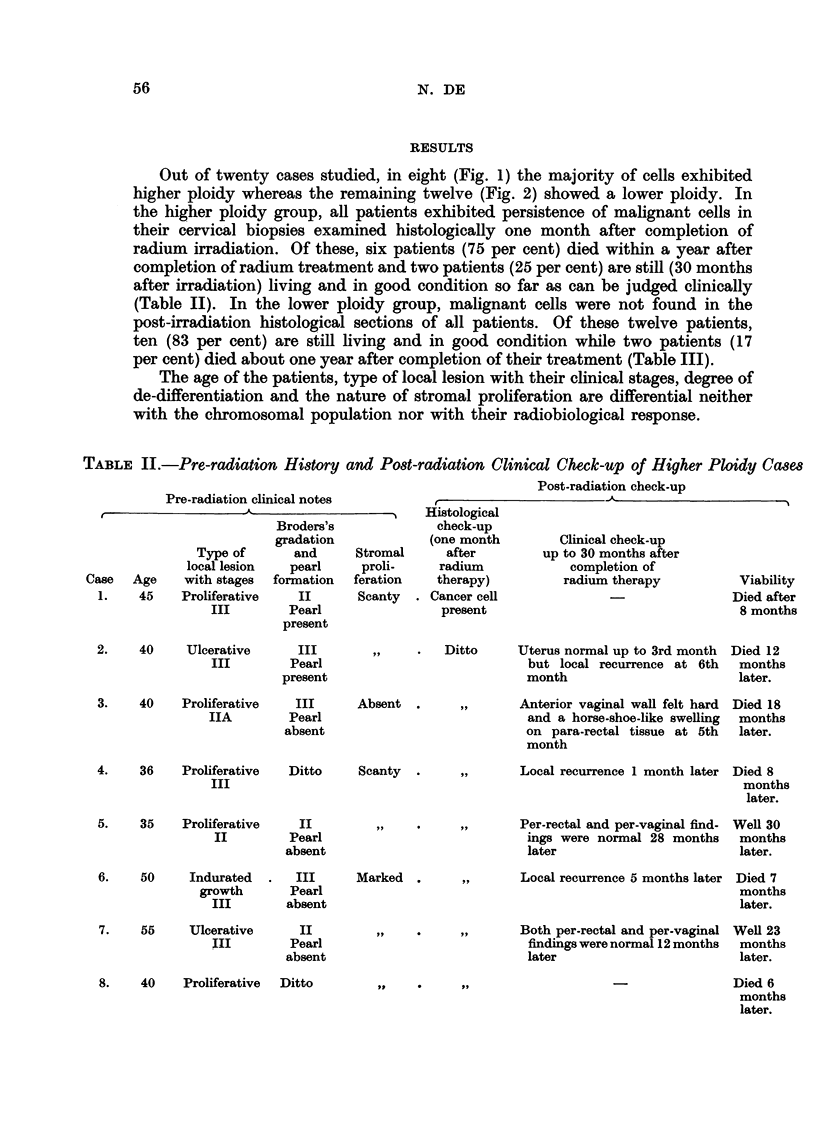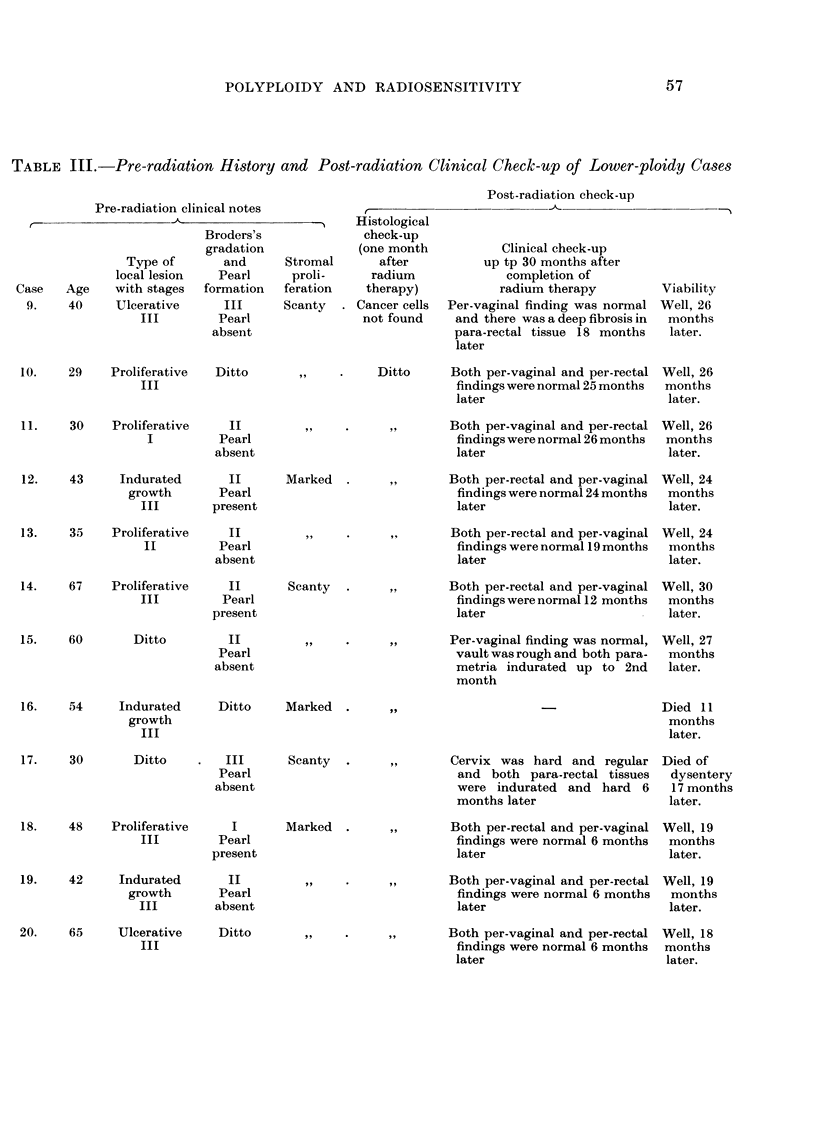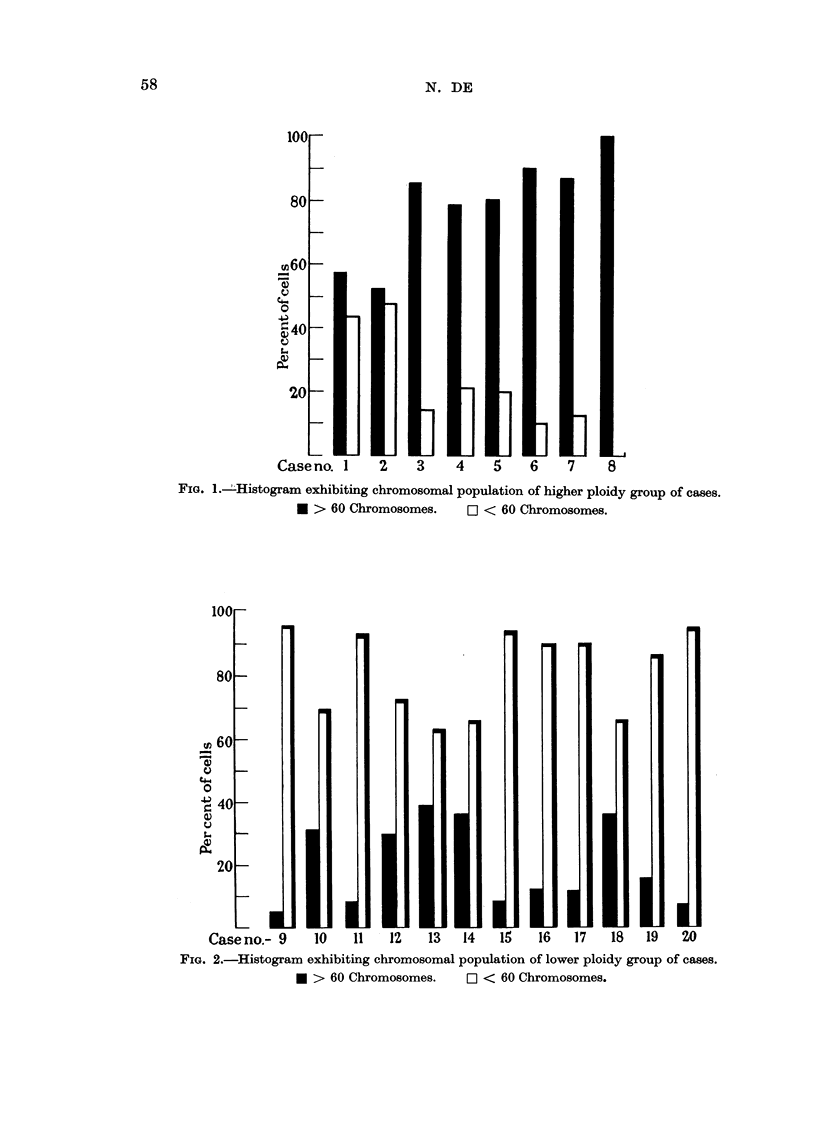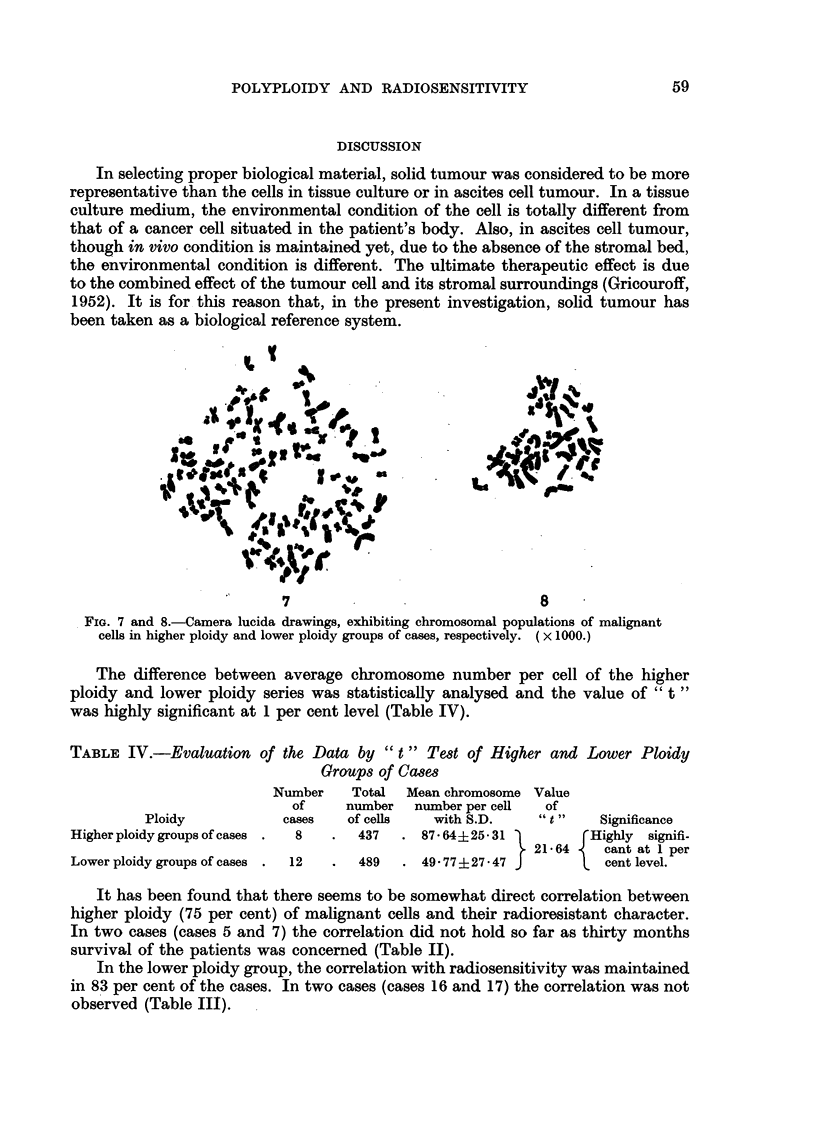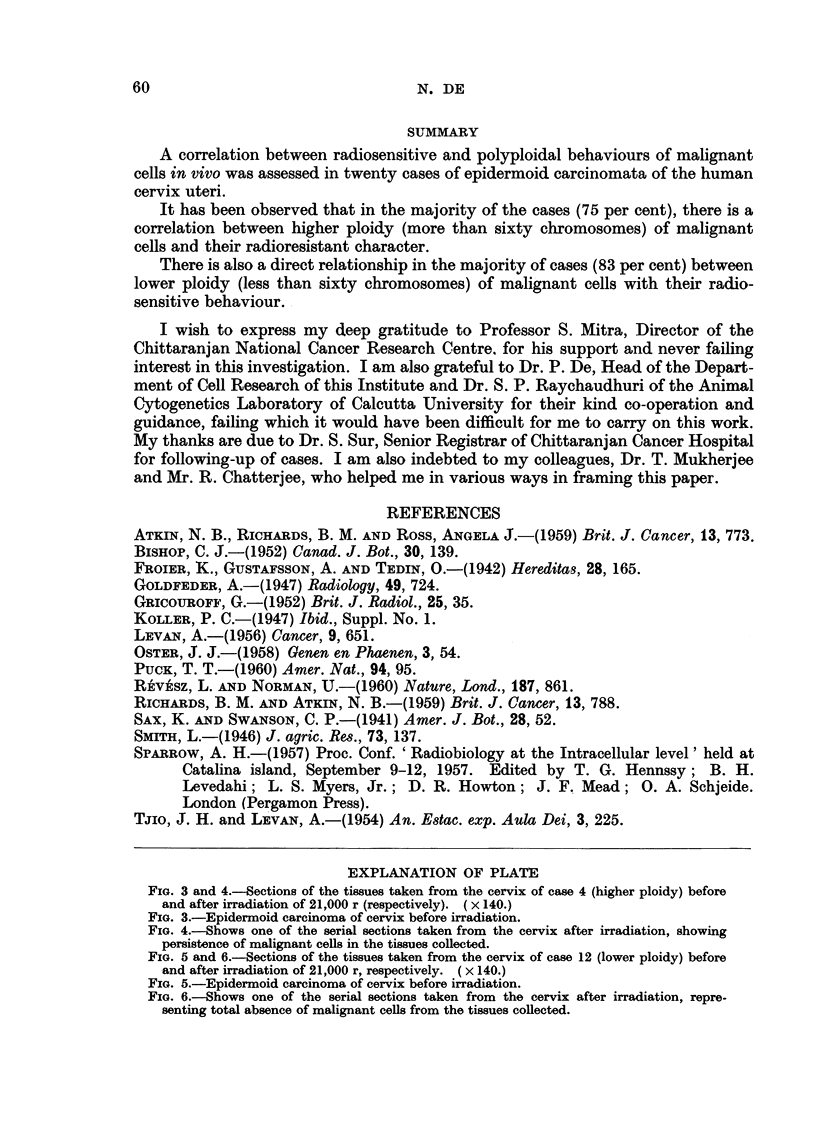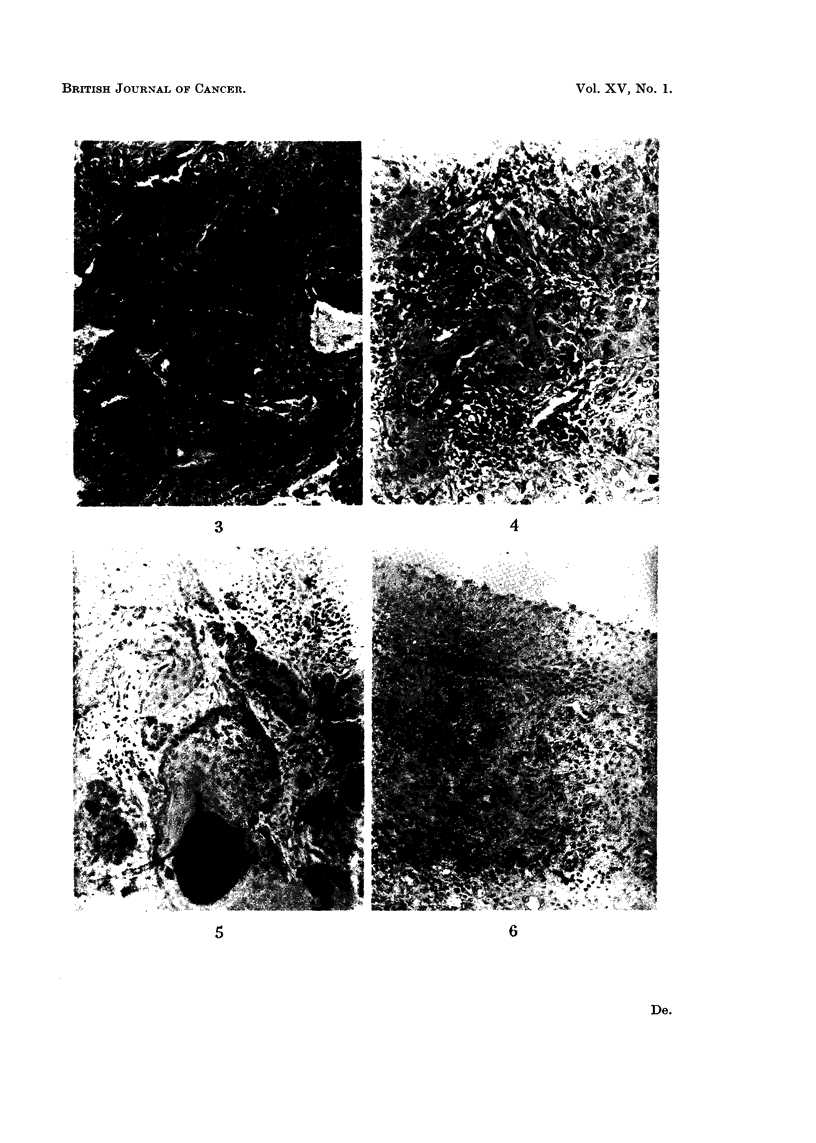# Polyploidy and Radiosensitive Behaviour of Human Malignant Cells In Vivo

**DOI:** 10.1038/bjc.1961.8

**Published:** 1961-03

**Authors:** N. De

## Abstract

**Images:**


### POLYPLOIDY AND RADIOSENSITIVE BEHAVIOUR OF HUMAN MALIGNANT CELLS *IN VIVO*

N. DE

*From the Department of Cell Research, Chittaranjan National Cancer Research Centre, Calcutta 26, India*

Received for publication January 10, 1961

IT is an established fact that malignant cells of the same type under the same radiation treatment react differently to the therapy. Goldfeder (1947) has shown that malignant cells of the same morphological type differ in radiosensitivity. Koller (1947) has also shown that the mean number of chromosome fragments per cell differs in cell samples taken from different regions of the same tumour after radiation treatment. In plant material, different authors have observed different types of correlation between polyploidy and radiosensitivity of normal cells. Smith (1946) and Bishop (1952) reported that the sensitivity of the polyploid cells is equal to that of diploids. Sax and Swanson (1941), Froier, Gustafsson and Tedin (1942), Sparrow (1957) and Oster (1958) are of the opinion that a high degree of radioresistance of normal cells is related to their high chromosome number. Puck (1960) demonstrated that *in vitro*, hyperploid and aneuploid normal human cells are more radioresistant than diploid cells. Révész and Norman (1960) have made a correlation study of chromosomal ploidy and radiosensitive behaviour of the ascites tumour cell population.

As the majority of malignant tissues of human patients are in solid form, so ultimate radiotherapeutic assessment should be made on solid tumour. Atkin, Richards and Ross (1959) and Richards and Atkin (1959) have made a study of some possible significance of radiotherapeutic effect and DNA content of different types of solid tumour arising from human uterus. They have suggested (Atkin *et al.*, 1959) that radioresistant cell strains are more often higher than diploid than near diploid. The present investigation was undertaken to study the relationship between the chromosomal population and radiosensitivity of epidermoid carcinoma cells of human cervix *in vivo*. The significance of this study is that here the nature of the biological material, i.e. the epidermoid carcinomatous condition, is kept constant.

### MATERIAL AND METHOD

Twenty patients with histological evidence of epidermoid carcinoma in the cervix uteri (without any previous history of radiotherapy) of different ages, stages, grades and stromal proliferation (Tables II and III) were selected from the out-patients department of the Chittaranjan Cancer Hospital. Tissues were collected from the cervices of the above patients for chromosomal study. Chromosome counting was made on aceto-orcein squash preparation made according to the method suggested by Tjio and Levan (1954), the tissue being fixed in 1 : 3

aceto-alcohol after pre-treatment in hypotonic saline solution (Levan, 1956) for thirty to forty-five minutes. Chromosome countings were made on temporary squash preparations.

The patients were treated with radium (50 mg. intra-uterine and 50 mg. in a vaginal box), and received, at the point from where the tissue for section was taken, a total dose of 21,000 r in three applications according to the modified Stockholm technique. The interval between the first and the second application was one week and between the second and third application was three weeks. Each time the radium was kept in position for 24 hours (total $2400 \times 3 = 7200$ mg. hours). About one month after completion of radium treatment, the dose received in the parametrium from radium application was supplemented by X-irradiation from a Million Volt X-ray unit (H.V.L. 3·3 mm. Pb 70 cm. F.S.D.). The total tumour dose delivered at each parametrium from the Million Volt unit was 3000 r.

One month after completion of radium irradiation, four or five pieces of tissue from different regions of cervix were again taken from each of the above selected patients for histological assessment of the radiotherapeutic effect. Tissues for histological study were fixed in Bouin's fluid and serial sections, which were stained in haematoxylin and eosin, were made through the whole tissue. These patients were kept under observation with repeated clinical examination every month (Tables II and III) up to a period of about thirty months from the date of the histological examination.

Cases in which the majority of the cells had an average chromosome number of more than sixty were taken as the higher ploidy group ; cases where the majority of the cells had an average chromosome number below sixty were taken as the lower ploidy group (Tables I, II and III).

TABLE I.—*Chromosomal Population of Malignant Cells of both Higher and Lower Ploidy Groups of Cases*

| Case Number | Number of cells studied | Percentage of cells with chromosome numbers | | Average chromosome number per cell containing | |
|---|---|---|---|---|---|
| | | >60 | <60 | >60 | <60 |
| 1 | 55 | 56·35 | 43·65 | 89·29±33·92 | 49·00±8·83 |
| 2 | 134 | 52·24 | 47·76 | 122·78±54·79 | 41·66±11·81 |
| 3 | 62 | 85·47 | 14·53 | 107·07±21·50 | 37·22±3·35 |
| 4 | 51 | 78·42 | 21·58 | 112·37±31·70 | 33·63±14·45 |
| 5 | 35 | 79·98 | 20·02 | 91·13±36·40 | 38·14±17·66 |
| 6 | 30 | 90·00 | 10·00 | 93·88±5·38 | 48·00±0 |
| 7 | 45 | 86·70 | 13·30 | 98·46±33·92 | 43·66±10·20 |
| 8 | 25 | 100·0 | 0 | 104·76±50·14 | 0±0 |
| 9 | 84 | 4·77 | 95·23 | 91·50±21·56 | 28·87±9·94 |
| 10 | 57 | 31·03 | 68·95 | 105·00±60·89 | 42·43±12·00 |
| 11 | 50 | 8·00 | 92·00 | 80·00±16·00 | 38·73±6·48 |
| 12 | 24 | 29·16 | 70·83 | 92·71±43·06 | 49·82±5·56 |
| 13 | 26 | 38·46 | 61·54 | 76·00±12·61 | 38·23±14·66 |
| 14 | 28 | 35·72 | 64·28 | 85·60±16·85 | 46·44±11·57 |
| 15 | 25 | 8·00 | 92·00 | 103·00±00·00 | 41·75±9·00 |
| 16 | 40 | 12·50 | 87·50 | 85·60±6·16 | 45·20±7·07 |
| 17 | 50 | 12·00 | 88·00 | 78·80±15·59 | 41·00±12·50 |
| 18 | 50 | 36·00 | 64·00 | 100·22±41·01 | 46·06±7·21 |
| 19 | 25 | 16·00 | 84·00 | 84·00±18·76 | 45·09±6·92 |
| 20 | 30 | 6·70 | 93·30 | 79·50±23·32 | 49·03±6·40 |

### RESULTS

Out of twenty cases studied, in eight (Fig. 1) the majority of cells exhibited higher ploidy whereas the remaining twelve (Fig. 2) showed a lower ploidy. In the higher ploidy group, all patients exhibited persistence of malignant cells in their cervical biopsies examined histologically one month after completion of radium irradiation. Of these, six patients (75 per cent) died within a year after completion of radium treatment and two patients (25 per cent) are still (30 months after irradiation) living and in good condition so far as can be judged clinically (Table II). In the lower ploidy group, malignant cells were not found in the post-irradiation histological sections of all patients. Of these twelve patients, ten (83 per cent) are still living and in good condition while two patients (17 per cent) died about one year after completion of their treatment (Table III).

The age of the patients, type of local lesion with their clinical stages, degree of de-differentiation and the nature of stromal proliferation are differential neither with the chromosomal population nor with their radiobiological response.

TABLE II.—*Pre-radiation History and Post-radiation Clinical Check-up of Higher Ploidy Cases*

| | | Pre-radiation clinical notes | | | Post-radiation check-up | | |
|---|---|---|---|---|---|---|---|
| Case | Age | Type of local lesion with stages | Broders's gradation and pearl formation | Stromal proliferation | Histological check-up (one month after radium therapy) | Clinical check-up up to 30 months after completion of radium therapy | Viability |
| 1. | 45 | Proliferative III | II Pearl present | Scanty | Cancer cell present | — | Died after 8 months |
| 2. | 40 | Ulcerative III | III Pearl present | ,, | Ditto | Uterus normal up to 3rd month but local recurrence at 6th month | Died 12 months later. |
| 3. | 40 | Proliferative IIA | III Pearl absent | Absent | ,, | Anterior vaginal wall felt hard and a horse-shoe-like swelling on para-rectal tissue at 5th month | Died 18 months later. |
| 4. | 36 | Proliferative III | Ditto | Scanty | ,, | Local recurrence 1 month later | Died 8 months later. |
| 5. | 35 | Proliferative II | II Pearl absent | ,, | ,, | Per-rectal and per-vaginal findings were normal 28 months later | Well 30 months later. |
| 6. | 50 | Indurated growth III | III Pearl absent | Marked | ,, | Local recurrence 5 months later | Died 7 months later. |
| 7. | 55 | Ulcerative III | II Pearl absent | ,, | ,, | Both per-rectal and per-vaginal findings were normal 12 months later | Well 23 months later. |
| 8. | 40 | Proliferative | Ditto | ,, | ,, | — | Died 6 months later. |

TABLE III.—*Pre-radiation History and Post-radiation Clinical Check-up of Lower-ploidy Cases*

| | Pre-radiation clinical notes | | | | Post-radiation check-up | | |
|---|---|---|---|---|---|---|---|
| Case | Age | Type of local lesion with stages | Broders's gradation and Pearl formation | Stromal proli-feration | Histological check-up (one month after radium therapy) | Clinical check-up up tp 30 months after completion of radium therapy | Viability |
| 9. | 40 | Ulcerative III | III Pearl absent | Scanty . | Cancer cells not found | Per-vaginal finding was normal and there was a deep fibrosis in para-rectal tissue 18 months later | Well, 26 months later. |
| 10. | 29 | Proliferative III | Ditto | ,, . | Ditto | Both per-vaginal and per-rectal findings were normal 25 months later | Well, 26 months later. |
| 11. | 30 | Proliferative I | II Pearl absent | ,, . | ,, | Both per-vaginal and per-rectal findings were normal 26 months later | Well, 26 months later. |
| 12. | 43 | Indurated growth III | II Pearl present | Marked . | ,, | Both per-rectal and per-vaginal findings were normal 24 months later | Well, 24 months later. |
| 13. | 35 | Proliferative II | II Pearl absent | ,, . | ,, | Both per-rectal and per-vaginal findings were normal 19 months later | Well, 24 months later. |
| 14. | 67 | Proliferative III | II Pearl present | Scanty . | ,, | Both per-rectal and per-vaginal findings were normal 12 months later | Well, 30 months later. |
| 15. | 60 | Ditto | II Pearl absent | ,, . | ,, | Per-vaginal finding was normal, vault was rough and both para-metria indurated up to 2nd month | Well, 27 months later. |
| 16. | 54 | Indurated growth III | Ditto | Marked . | ,, | — | Died 11 months later. |
| 17. | 30 | Ditto . | III Pearl absent | Scanty . | ,, | Cervix was hard and regular and both para-rectal tissues were indurated and hard 6 months later | Died of dysentery 17 months later. |
| 18. | 48 | Proliferative III | I Pearl present | Marked . | ,, | Both per-rectal and per-vaginal findings were normal 6 months later | Well, 19 months later. |
| 19. | 42 | Indurated growth III | II Pearl absent | ,, . | ,, | Both per-vaginal and per-rectal findings were normal 6 months later | Well, 19 months later. |
| 20. | 65 | Ulcerative III | Ditto | ,, . | ,, | Both per-vaginal and per-rectal findings were normal 6 months later | Well, 18 months later. |

Fig. 1.—Histogram exhibiting chromosomal population of higher ploidy group of cases.
■ > 60 Chromosomes. □ < 60 Chromosomes.

Fig. 2.—Histogram exhibiting chromosomal population of lower ploidy group of cases.
■ > 60 Chromosomes. □ < 60 Chromosomes.

### DISCUSSION

In selecting proper biological material, solid tumour was considered to be more representative than the cells in tissue culture or in ascites cell tumour. In a tissue culture medium, the environmental condition of the cell is totally different from that of a cancer cell situated in the patient's body. Also, in ascites cell tumour, though *in vivo* condition is maintained yet, due to the absence of the stromal bed, the environmental condition is different. The ultimate therapeutic effect is due to the combined effect of the tumour cell and its stromal surroundings (Gricouroff, 1952). It is for this reason that, in the present investigation, solid tumour has been taken as a biological reference system.

FIG. 7 and 8.—Camera lucida drawings, exhibiting chromosomal populations of malignant cells in higher ploidy and lower ploidy groups of cases, respectively. (×1000.)

The difference between average chromosome number per cell of the higher ploidy and lower ploidy series was statistically analysed and the value of " t " was highly significant at 1 per cent level (Table IV).

TABLE IV.—*Evaluation of the Data by "t" Test of Higher and Lower Ploidy Groups of Cases*

| Ploidy | Number of cases | Total number of cells | Mean chromosome number per cell with S.D. | Value of "*t*" | Significance |
|---|---|---|---|---|---|
| Higher ploidy groups of cases | 8 | 437 | 87·64±25·31 | 21·64 | Highly significant at 1 per cent level. |
| Lower ploidy groups of cases | 12 | 489 | 49·77±27·47 | | |

It has been found that there seems to be somewhat direct correlation between higher ploidy (75 per cent) of malignant cells and their radioresistant character. In two cases (cases 5 and 7) the correlation did not hold so far as thirty months survival of the patients was concerned (Table II).

In the lower ploidy group, the correlation with radiosensitivity was maintained in 83 per cent of the cases. In two cases (cases 16 and 17) the correlation was not observed (Table III).

SUMMARY

A correlation between radiosensitive and polyploidal behaviours of malignant cells *in vivo* was assessed in twenty cases of epidermoid carcinomata of the human cervix uteri.

It has been observed that in the majority of the cases (75 per cent), there is a correlation between higher ploidy (more than sixty chromosomes) of malignant cells and their radioresistant character.

There is also a direct relationship in the majority of cases (83 per cent) between lower ploidy (less than sixty chromosomes) of malignant cells with their radiosensitive behaviour.

I wish to express my deep gratitude to Professor S. Mitra, Director of the Chittaranjan National Cancer Research Centre, for his support and never failing interest in this investigation. I am also grateful to Dr. P. De, Head of the Department of Cell Research of this Institute and Dr. S. P. Raychaudhuri of the Animal Cytogenetics Laboratory of Calcutta University for their kind co-operation and guidance, failing which it would have been difficult for me to carry on this work. My thanks are due to Dr. S. Sur, Senior Registrar of Chittaranjan Cancer Hospital for following-up of cases. I am also indebted to my colleagues, Dr. T. Mukherjee and Mr. R. Chatterjee, who helped me in various ways in framing this paper.

### REFERENCES

Atkin, N. B., Richards, B. M. and Ross, Angela J.—(1959) *Brit. J. Cancer*, **13**, 773.
Bishop, C. J.—(1952) *Canad. J. Bot.*, **30**, 139.
Froier, K., Gustafsson, A. and Tedin, O.—(1942) *Hereditas*, **28**, 165.
Goldfeder, A.—(1947) *Radiology*, **49**, 724.
Gricouroff, G.—(1952) *Brit. J. Radiol.*, **25**, 35.
Koller, P. C.—(1947) *Ibid.*, Suppl. No. 1.
Levan, A.—(1956) *Cancer*, **9**, 651.
Oster, J. J.—(1958) *Genen en Phaenen*, **3**, 54.
Puck, T. T.—(1960) *Amer. Nat.*, **94**, 95.
Révész, L. and Norman, U.—(1960) *Nature, Lond.*, **187**, 861.
Richards, B. M. and Atkin, N. B.—(1959) *Brit. J. Cancer*, **13**, 788.
Sax, K. and Swanson, C. P.—(1941) *Amer. J. Bot.*, **28**, 52.
Smith, L.—(1946) *J. agric. Res.*, **73**, 137.
Sparrow, A. H.—(1957) Proc. Conf. ' Radiobiology at the Intracellular level ' held at Catalina island, September 9–12, 1957. Edited by T. G. Hennssy ; B. H. Levedahi ; L. S. Myers, Jr. ; D. R. Howton ; J. F. Mead ; O. A. Schjeide. London (Pergamon Press).
Tjio, J. H. and Levan, A.—(1954) *An. Estac. exp. Aula Dei*, **3**, 225.

---

### EXPLANATION OF PLATE

Fig. 3 and 4.—Sections of the tissues taken from the cervix of case 4 (higher ploidy) before and after irradiation of 21,000 r (respectively). (×140.)

Fig. 3.—Epidermoid carcinoma of cervix before irradiation.

Fig. 4.—Shows one of the serial sections taken from the cervix after irradiation, showing persistence of malignant cells in the tissues collected.

Fig. 5 and 6.—Sections of the tissues taken from the cervix of case 12 (lower ploidy) before and after irradiation of 21,000 r, respectively. (×140.)

Fig. 5.—Epidermoid carcinoma of cervix before irradiation.

Fig. 6.—Shows one of the serial sections taken from the cervix after irradiation, representing total absence of malignant cells from the tissues collected.